# Aptamers: Promising Tools for Cancer Diagnosis and Therapy

**DOI:** 10.3390/cancers10050132

**Published:** 2018-05-03

**Authors:** Laura Cerchia

**Affiliations:** Istituto per l'Endocrinologia e l'Oncologia Sperimentale “G. Salvatore” (IEOS), Consiglio Nazionale delle Ricerche, Via S. Pansini 5, 80131 Naples, Italy; cerchia@unina.it; Tel: +39-081-545-5751

The most common approaches to cancer treatment have been, for decades, based on surgical excision, radio- and/or chemotherapy, which, in spite of their modest survival benefits, still encounter several limitations, in part due to their lack of specificity. Conventional chemotherapeutics usually kill highly proliferative cells, without distinguishing between cancer cells and rapidly dividing normal cells, thus leading to severe toxic side effects. Moreover, they are also associated with inefficient delivery to tumor tissues and low intratumoral accumulation, so high doses are required to obtain therapeutic efficacy. In the last decade, significant advancements in the knowledge of molecular mechanisms driving malignant progression have been revolutionizing approaches to cancer treatment. Precision cancer medicines that act specifically at the diseased site, minimizing drug uptake by non-malignant cells, are now achieving significant progress, especially through the development of active targeting agents.

Aptamers, isolated by the Systematic Evolution of Ligands by EXponential enrichment (SELEX) process, are highly structured, short, single-stranded oligonucleotides that, because of their complex tridimensional shapes, interact at high affinity and selectivity with their targets [1]. For their mode of action, aptamers are also called chemical antibodies; however, unlike antibodies, they exhibit high tissue penetration due to their small size and are poorly immunogenic if at all. Furthermore, aptamers are characterized by ready synthesis and chemical modifications designed to improve their stability, reduce toxicity, and allow conjugation with other chemical entities (including therapeutics and molecular imaging probes). Since their first discovery in 1990, aptamers have spawned a productive academic and commercial industry. Still, the translation of pre-clinical research on aptamers into clinical use as cancer therapeutics is more advanced than generally thought, with two aptamers, the anti-nucleolin AS1411 aptamer [2] and the anti-CXCL12 NOX-A12 aptamer [3], currently under evaluation as anti-cancer therapies.

Researchers worldwide are attempting to the generation of aptamers to use as recognition elements in a number of applications, including cancer biomarkers discovery, targeted therapy, in vitro diagnosis and in vivo imaging modalities, and several key articles have recently appeared, indicating that this is a rapid evolving field [4]. 

The six review articles in this Special Issue of *Cancers* provide an updated overview of the SELEX technology and highlight the latest concepts about the use of aptamers as diagnostic and therapeutic agents, and as active targeting agents to enhance the therapeutic efficacy of nanomedicines in cancer. Each article focuses on unique aspects of aptamer-related research, thus collectively providing a valuable update to this field and inspiring researchers to investigate novel strategies for cancer targeting, delivery and diagnosis. 

The article by Morita et al. [5] offers an exhaustive review of the use of aptamers as therapeutic agents in cancers, with an emphasis on challenges and possible solutions towards their clinical application. The authors critically discuss the unfavorable pharmaceutical properties of aptamers, mostly due to their susceptibility to nuclease degradation and renal excretion, and the methods used to face these challenges. Several examples are reported in which sophisticated chemistries, applicable at different positions of the nucleic acid structure, confer high nuclease degradation resistance, still maintaining, or even increasing, affinity and specificity. Still, approaches to overcome rapid renal excretion, consisting of augmentation of the aptamer’s overall size through conjugation with high molecular weight moieties, are reviewed, highlighting the pros and cons of the used moiety. Furthermore, the issue of the safety of aptamers is addressed, showing that intravenously administered aptamers are generally well tolerated, even if a few adverse effects related to dose and sequence have been reported. In this regard, a paragraph discusses preclinical studies with bi-specific aptamers, which are capable of widening the therapeutic window and reducing the toxicity of a given strategy. It is also discussed how antidote aptamers, a strategy to control the action time of therapeutic aptamers using the corresponding complementary sequence, were revealed to be an efficacious tool to balance therapeutic efficacy and toxicity in animal models. As reviewed by the authors, aptamers have been reported to target a broad range of molecules, with crucial roles in cancer, including cancer cell proliferation, cell homing, apoptosis suppression, metastasis, impairment of T-cell cytotoxicity, and angiogenesis. These molecules are under pre-clinical investigation for anti-cancer therapy.

The article by Tan and co-workers [6] primarily highlights the utility of SELEX on living cells to generate aptamers against cell-surface proteins, discussing various applications of selected aptamers in areas related to cancer, including in vivo imaging, targeted drug delivery and therapy. The authors discuss the use of aptamers as targeting components instead of peptides or proteins in targeted therapy approaches, including phototherapy, gene therapy and chemotherapy, and the possibility of coupling the cell-SELEX technique with the discovery of new tumor biomarkers, indicating that the selected aptamers may significantly contribute to the broad development of personalized cancer medicine. In these fields, the aptamers generated in the authors’ lab have been discussed as examples of high versatility of aptamers for different uses. However, despite great progress in aptamer-related research, the authors highlight how some limitations remain. They present to the readers the major existing difficulties with which researchers in this field are dealing, such as the need to reduce the degree of randomness of the cell-SELEX process and the time required to identify aptamers specific to different cancer-related samples, as well as optimization of aptamer’s efficacy, stability and half-life, especially in complex tumor microenvironments. 

The use of both DNA and RNA aptamers for specific delivery of chemotherapeutics to tumor cells, both in vitro and in animal models, has been investigated by the group of Shigdar using an epithelial cell adhesion molecule (EpCAM), a type 1 transmembrane glycoprotein highly expressed in epithelial cancers, as a targeted protein. In the present article [7], the authors underline how EpCAM, one of the first cancer biomarkers to be discovered, has been intensively investigated as a target for both diagnosis and therapy, and present a clear description of anti-cancer approaches, moving from antibody-mediated immunotherapies to aptamer-mediated targeted chemotherapies. By reading this article, we learn that, while monoclonal antibodies have been investigated against EpCAM for almost 40 years as a primary or adjuvant therapy, they failed to deliver on the initial promises. The efficacy of antibodies is limited, due either to the lack of solid tumor penetration or to the need for active engagement of CD16-positive immune cells. The authors address the reasons of limited therapeutic efficacy of EpCAM antibodies and propose alternative targeting options including aptamers. Importantly, the performance of an EpCAM aptamer was superior to that of an EpCAM antibody in theranostic applications, displaying better tumor penetration, more homogeneous distribution and longer retention in the tumor. 

The review by Rossi, Zhou and colleagues [8] begins with a thoughtful summary of the history and recent advances in SELEX technology against purified proteins (protein-based SELEX), cultured cells (whole cell-based SELEX) and targets within live animals (live animal-based SELEX). Then, they discuss the use of high-throughput sequencing technology and bioinformatics analysis combined with SELEX (HT-SELEX) to identify high-affinity aptamers coming from the selection. The authors also review the evidences supporting the usefulness of aptamers in cancer detection approaches, particularly in electrochemical aptasensors, fluorescent aptasensors, and nanoparticle-conjugated aptamers. With an emphasis on advances from the past year, they summarize different systems to detect cancer cells and biomarkers, highlighting highly specific aptamers that have been proven to enable earlier and more sensitive detection of cancers that are difficult to diagnose at an early stage. Still, innovative aptamer-based approaches to detect metastasis, by directly targeting metastasis-associated proteins or circulating tumor cells, and to hit target for multiple cancers, have been summarized. A section of this review is dedicated to highlighting several recent studies investigating aptamers endowed with anticancer activity in vivo. Also, the article provides an exhaustive description of current approaches and recent progress that has been achieved in using aptamers as targeting carriers, including the direct conjugation of aptamers to small chemical drugs or therapeutic oligonucleotides and their use to deliver and functionalize nanomaterials. 

The last two articles focus on the use of aptamers in diagnostic applications. Musumeci et al. [9] report an updated overview on aptamer-based fluorescent biosensors, designed to recognize significant cancer biomarkers both in soluble and in cancer cell membrane-bound form. The authors emphasize that fluorescence is one of the most commonly used optical techniques and has been widely applied to produce aptasensors because of its good sensitivity, efficiency, ease of application, low cost and non-invasive nature. Antibodies are widely used to detect and quantify antigens; however, while the fluorescence signal of fluorescently labeled antibodies generally remains the same whether or not the antibody is bound to the target, conformational transitions of the aptamer upon ligand binding can be conveniently monitored using covalently attached luminescent labels or non-covalent luminescent probes. The authors provide several examples in which, upon target binding, conformational changes of the aptamer are transduced in a fluorescent signal using both aptamers covalently labeled with fluorophores and label-free aptamers, and review different sensing strategies (“signal-on”, “signal-off” mode, etc.) employed for quantitative measurement of the target concentration. Starting from aptasensors for thrombin, representing the most studied protein for aptamer recognition, typically exploited as a proof of concept, the authors elegantly discuss a number of aptamer-based fluorescent systems developed for the selective detection of important targets implicated in tumorigenesis, including platelet-derived growth factor, vascular endothelial growth factor, angiogenin, mucin, elastase, protein tyrosine kinase 7 and lysozyme. Still, a paragraph summarizes fluorescent aptamers that have been developed for the in vitro and in vivo imaging of cancer cells and tissues and their use for diagnosis and intraoperative surgery.

Zamay et al. [10] discuss the usefulness of oligonucleotide aptamers for biomarker discovery, with a focus on lung cancer. As reported by the authors, the high mortality of lung cancer is largely a consequence of patients presenting late when the cancer is already at an advanced stage; early diagnosis of this disease is an urgent need. Unfortunately, the high heterogeneity of each histological lung cancer type and the absence of well-defined molecular biomarkers able to distinguish different types make lung cancer diagnosis rather difficult. The authors give information about the classification of various types of lung cancer using known biomarkers. Importantly, they suggest the use of a panel of eight tumor-associated biomarkers (CEA, CYFRA21-1, ProGRP, CEA, PSF3, MUC, SCCA, and SST) to differentiate lung cancer histological types and define the metastasis rate. Furthermore, they provide an original discussion of how aptamer-based detection of lung cancer biomarkers may guide accurate diagnosis in addition to conventional biomarker discovery methods. Aptamers may serve as recognition elements in several diagnostic applications, such as electrochemical detection of oncoproteins present in the blood plasma, fluorescence detection and capture of circulating tumor cells and aptamer-based staining of lung cancer tissues. Studies performed by the authors proved the feasibility of aptamers in these fields. Indeed, they successfully applied DNA aptamers, selected against lung adenocarcinoma cells derived from postoperative tissues, to the detection of lung cancer biomarkers, including laminin, vimentin, defensin and tubulin, by aptamer-based affinity enrichment methods. These molecules were able to detect circulating tumor cells in human blood and characterize the histological structure of lung adenocarcinoma in aptahistochemistry analyses. 

In summary, this Special Issue provides a well-rounded overview of current aptamer-based strategies applied to cancer biology, diagnosis and therapy. Much work is needed to overcome the limitations and difficulties still encountered in this field, but we believe in the promising future of aptamers, and hope the related research work can be rapidly implemented.
